# Quantification of joint mobility limitation in adult type 1 diabetes

**DOI:** 10.3389/fendo.2023.1238825

**Published:** 2023-11-06

**Authors:** Sanat Phatak, Pranav Mahadevkar, Kaustubh Suresh Chaudhari, Shreya Chakladar, Swasti Jain, Smita Dhadge, Sarita Jadhav, Rohan Shah, Aboli Bhalerao, Anupama Patil, Jennifer L. Ingram, Pranay Goel, Chittaranjan S. Yajnik

**Affiliations:** ^1^ Diabetes Unit, King Edward Memorial (KEM) Hospital Research Centre, Pune, India; ^2^ Department of Musculoskeletal Radiology, Star Imaging and Research Centre, Pune, India; ^3^ Department of Biology, Indian Institute of Science Education and Research, Pune, India; ^4^ Division of Pulmonary, Allergy and Critical Care, Department of Medicine, Duke University Medical Center, Durham, NC, United States

**Keywords:** magnetic resonance imaging (MRI), outcome measure (healthcare), tenosynovitis, metacarpophalangeal (MCP) joint, limited joint mobility (LJM), stiffness

## Abstract

**Aims:**

Diabetic cheiroarthropathies limit hand mobility due to fibrosis and could be markers of a global profibrotic trajectory. Heterogeneity in definitions and lack of a method to measure it complicate studying associations with organ involvement and treatment outcomes. We measured metacarpophalangeal (MCP) joint extension as a metric and describe magnetic resonance (MR) imaging determinants of MCP restriction.

**Methods:**

Adults with type 1 diabetes were screened for hand manifestations using a symptom questionnaire, clinical examination, and function [Duruoz hand index (DHI) and grip strength]. Patients were segregated by mean MCP extension (<20°, 20°–40°, 40°–60°, and >60°) for MR imaging (MRI) scanning. Patients in the four groups were compared using ANOVA for clinical features and MRI tissue measurements (tenosynovial, skin, and fascia thickness). We performed multiple linear regression for determinants of MCP extension.

**Results:**

Of the 237 patients (90 men), 79 (33.8%) with cheiroarthropathy had MCP extension limitation (39° versus 61°, p < 0.01). Groups with limited MCP extension had higher DHI (1.9 vs. 0.2) but few (7%) had pain. Height, systolic blood pressure, and nephropathy were associated with mean MCP extension. Hand MRI (n = 61) showed flexor tenosynovitis in four patients and median neuritis in one patient. Groups with MCP mobility restriction had the thickest palmar skin; tendon thickness or median nerve area did not differ. Only mean palmar skin thickness was associated with MCP extension angle on multiple linear regression.

**Conclusion:**

Joint mobility limitation was quantified by restricted mean MCP extension and had structural correlates on MRI. These can serve as quantitative measures for future associative and interventional studies.

## Highlights

What is already known:

- Diabetic cheiroarthropathies (limited joint mobility, carpal tunnel syndrome, flexor tenosynovitis, and carpal tunnel syndrome) limit hand function in type 1 diabetes.
- Associations with vascular complications are inconsistent, and a relationship with internal organ fibrosis is not known.
- A lack of a method to measure the amount of hand fibrosis contributes to difficulties in establishing associations.

What is the key question:

Is average metacarpophalangeal (MCP) joint extension a marker of joint stiffness in type 1 diabetes?

What are the new findings:

- Cheiroarthropathies limit extension at the MCP joint, despite being rarely symptomatic.
- Mean MCP extension was associated with other indicators of tissue stiffness such as blood pressure and proteinuria.
- MCP extension limitation has structural correlates on magnetic resonance imaging, chiefly skin thickening.

How this study might affect research:

- Mean MCP extension angle in the clinic, and standardized measurements of tissue thickness on hand MRI could both potentially be used as metrics of hand fibrosis in diabetes. With further validation, they could be used for associations with internal organ fibrosis as well as outcome measures for anti-fibrotic therapies.

## Introduction

Limitation of joint mobility in type 1 diabetes was described by Rosenbloom in 1974 (1). “Diabetic cheiroarthropathies,” hand conditions with a higher prevalence in diabetes, include limited joint mobility (LJM), carpal tunnel syndrome (CTS), flexor tenosynovitis (FTS), and Dupuytren’s contracture (DC) (2–4). Prevalence in type 1 diabetes ranges widely (8% to 66% LJM, 30% CTS, 28% FTS, and 9% DC) (2, 5–8). Their presence correlates with some but not all microvascular complications; these associations have often not been replicated (9). This variability owes to a heterogeneity in definitions and diagnostic methods in addition to population differences.

All cheiroarthropathies are fibrotic on histomorphology; biopsies demonstrate excessive collagen deposition in periarticular connective tissue (10). Irrespective of diabetes, hand tissue fibrosis in DC is associated internal organ fibrosis, especially the liver (11). Diabetes is associated with increased organ fibrosis, affecting the kidneys, heart, and liver, that leads to considerable morbidity and mortality (12). With a perceived common pathology, it is tempting to speculate that hand fibrosis externally reflects a more global profibrotic trajectory (13, 14). In such a situation, examining the hand could serve as a useful clinical biomarker to select out patients for internal organ fibrosis. However, establishing such associations is currently hampered by a lack of consensus in measuring the severity of hand involvement.

It is only in severe cases that flexion contractures ensue, affecting professional and self-care activities, as well as metabolic complications (15–17). However, an ideal metric should be able to detect subclinical involvement, encapsulate all the differing manifestations, and be easy and reproducible. Measurement of joint mobility has been previously used, and subjects with type 1 diabetes had limitations in wrist and interphalangeal (IP) flexion (18). All cheiroarthropathies result in a preferential inelasticity of structures on the palmar aspect of the hand, thus principally limiting finger extension. This typically involves all fingers in LJM, and one or two in FTS and DC. We therefore explored the utility of measuring mean metacarpophalangeal (MCP) extension as a measure of joint stiffness in diabetes in a cohort of adult patients with type 1 diabetes. In a subset of patients chosen across the spectrum of MCP extension limitation, we report magnetic resonance imaging (MRI) findings, including tissue thickening and contributors to joint stiffness.

## Patients and methods

### Patients and clinical setting

The study was conducted at the Diabetes Unit, KEM Hospital Research Centre, a tertiary care specialized unit in Pune, India. We screened consecutive adult patients (>18 years of age) with type 1 diabetes, from March 2021 to December 2022. We excluded pregnant patients, those who needed hospital admission, and those with hand trauma or concurrent active inflammatory arthritis that precluded hand examination. We recorded age, gender, education, and occupation. Manual work was classified as agriculture work, manual labor, or operating heavy machinery for more than 6 h a day; keyboard work if usage was more than 6 h a day. Smoking (current, previous, and never) and alcohol habits were recorded.

We extracted date of diagnosis, insulin compliance, and micro- and macro-vascular complications from patient files. Retinopathy was diagnosed on fundus photographs, nephropathy with proteinuria and/or end stage renal disease, neuropathy on biothesiometry, and clinical composite score. Common comorbidities (hypertension and hypothyroidism) and conditions or medications that could contribute to fibrosis (systemic sclerosis, skin disease involving the palms, malignancy, controlled inflammatory arthritis; methotrexate, amiodarone, aspirin, statins, and anti-epileptics) were recorded. Height, weight, and blood pressure were recorded using standard procedures. We looked for keloid scars, lipo-hypertrophy, or atrophy at insulin injection sites. We used skin autofluorescence as an indirect measurement of advanced glycation end-product (AGE) deposition, using the AGE reader on the non-dominant forearm in standard light conditions (19).

### Quantitative measures of hand manifestations

We screened for hand involvement using a structured history and measurements by trained research staff. The musculoskeletal history included presence of palmar pain, symptoms of compressive neuropathy (sensory and motor), grip difficulty, finger triggering, and perceived stiffness and tightness of palmar skin. We used the Duruoz Hand Index (DHI) that assesses activity limitation in 18 daily activities on a visual analogue scale, for hand function (20).

**Figure 1:**
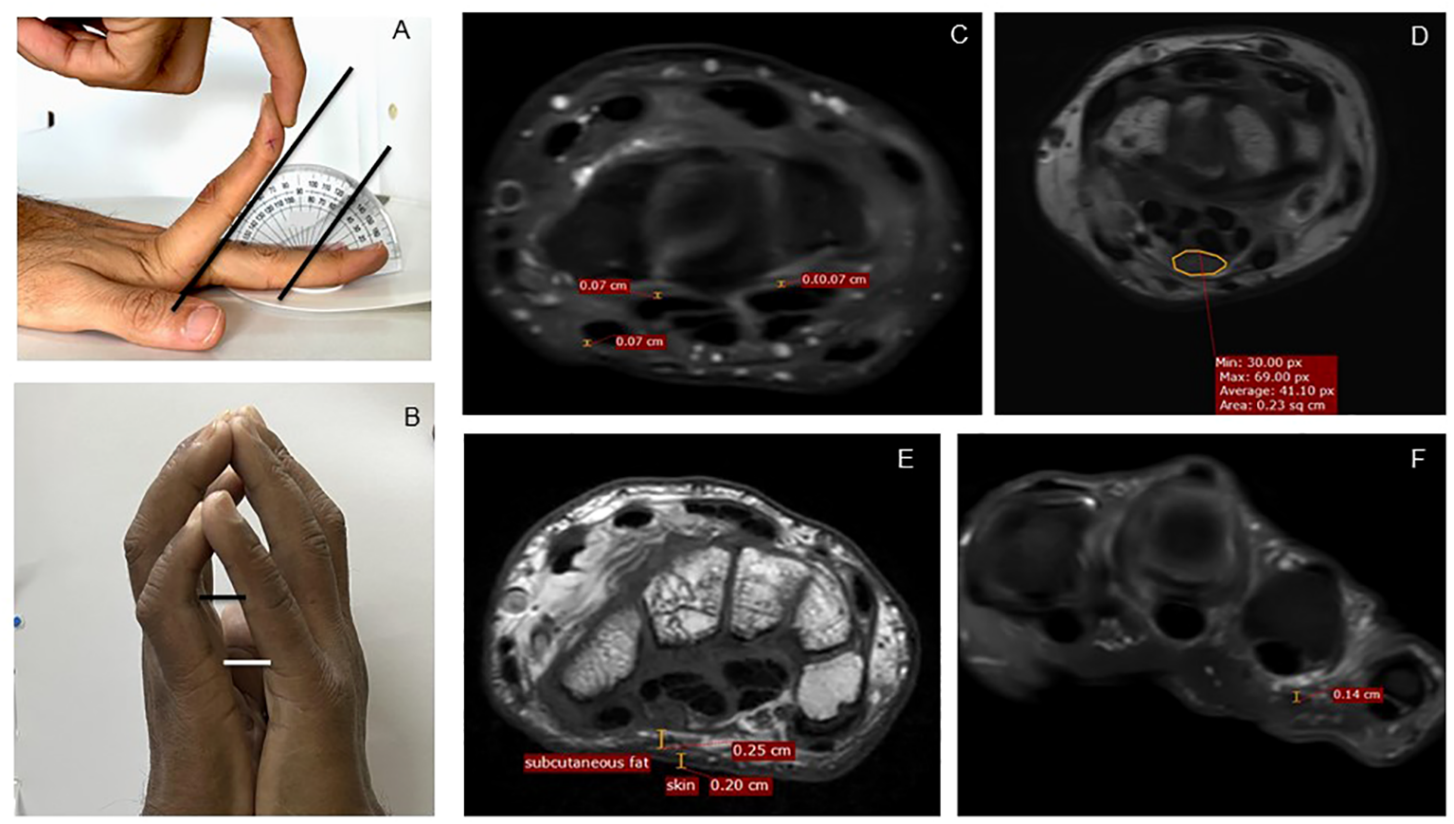
Measurement methodology, clinical and MRI. **(A)** Measurement of maximum passive extension at MCP joint of the left second finger, here showing 50° extension. **(B)** Measurements of distance between the fifth proximal interphalangeal joints and the fifth MCP joints as a quantification of the prayer sign. **(C)** Measurement of tenosynovial thickness at the level of carpal tunnel. **(D)** Measurement of median nerve area at the level of the carpal tunnel. **(E)** Measurement of skin and subcutaneous fat. **(F)** Measurement of palmar fascia overlying the fourth flexor tendon on PDFS images. MRI, magnetic resonance imaging; PDFS, proton density fat saturation.

We recorded maximum possible passive extension at the MCP joint (second to fifth of both hands) until restriction or pain, with the palm approximated on a flat surface, using a protractor (**Figure 1A**). Mean passive MCP extension was calculated for each hand as the average of four finger extension angles. A prayer sign was defined as a visible gap between the two palms with an inability to approximate them fully. We measured the distance between the two fifth MCP and fifth proximal IP (PIP) joints, viewed from the ulnar aspect (**Figure 1B**). The presence of flexor tendon thickening, nodularity, triggering, and crepitus was noted. For CTS, we examined for the Tinel sign, the Phalen sign, and sensation in the median nerve distribution, recorded as normal, reduced, or absent. Hand grip strength was measured as an average of three readings using a Jamar hand dynamometer (Patterson Medical, Warrenville, IL). A physician (rheumatologist or diabetologist) examined all patients independently without access to the above measurements and assessed if one or more hand manifestation was present (LJM, FTS, CTS, and DD). Inter-rater reliability between two physicians (SD and SP), seen in 30 patients, was 0.88.

### Selection of patients for MRI scans

Non-dominant hand mean MCP extension was used as a metric of joint mobility limitation to segregate patients into 20° bins (0°–20°, 20°–40°, 40°–60°, and >60°). Consecutive patients within each group were approached for MRI with a plan of including 15 per group, regardless of physician opinion.

Unless manifestations were unilateral, an MRI of the non-dominant hand was performed at the Star Imaging Research Centre using a 3T MRI Superconducting system with eight-channel extremity coil (Ingenia Release 5, Philips Healthcare, Amsterdam, The Netherlands). Consenting patients were invited to the Diabetes Unit, where earlier clinical findings were confirmed; contraindications to MRI were excluded (metallic bone; cardiac, cochlear, or dental implants; metallic intrauterine contraceptive devices; pregnancy; and claustrophobia). Two patients were on insulin pumps, and both the pump and sensor-transmitters were removed for the scan. Random plasma glucose measurements were performed, and the diabetologist (SD) managed insulin dosages accordingly, to prevent hypoglycemia while inside the MRI scanner.

### MR sequences and measurements

The following MR sequences were performed: axial and coronal T2 weighted images, and pre-contrast fat-saturated T1-weighted axial coronal with fat saturation. All MRI scans were read by the same musculoskeletal radiologist (PM) who provided a qualitative report (altered signals, thickening, and edema) on the status of bones, tendons, joints, median nerve, and other salient findings. Quantitative measurements were performed by one of two trained researchers (SC and SJ) on axial images, in addition to the radiologist (PM). The readers had good internal consistency (Cronbach alpha of 0.95) and agreement with each other (correlation coefficient of 0.78). Measurements included tenosynovial thickness for the four flexor digitorum longus tendons and the flexor hallucis longus tendon, at four levels: MCP joint, proximal phalanx midpoint, metacarpal bone midpoint, and carpal tunnel midpoint (**Figure 1C**). Mean tenosynovial thickness was calculated as average of these 20 data points. In addition, the thickness at a point of visually perceived maximum thickness for each tendon sheath was recorded. Skin and subcutaneous tissue thickness was measured at four points at the level of MCP joint and carpal tunnel, respectively, and an average was calculated (**Figure 1E**). Palmar fascia was visible only when thickened; it was measured at the site of thickening and was considered zero in others (**Figure 1F**). The median nerve cross-sectional area was measured at the level of the carpal tunnel outlet and median nerve signal abnormalities were noted (**Figure 1D**). An instinctive “total hand fibrosis” score was calculated by adding mean tenosynovial thickness, palmar fascia thickness, and palmar skin thickness. A similar score was also calculated using Z-scores of mean tenosynovial thickness, palmar fascia thickness, and palmar skin thickness (total hand fibrosis Z-score).

### Statistical analysis

Data are presented as mean (standard deviation) and median (interquartile range) as appropriate. Patients with cheiroarthropathy were compared with those without, using T-tests. Patients with different levels of MCP extension (0°–20°, 20°–40°, 40°–60°, and >60°) were compared with each other with respect to demographics, diabetes characteristics, and MRI characteristics using ANOVA; we report p-value for the trend, with <0.05 considered significant. Univariate linear regression adjusted for age and sex was used for determinants of hand stiffness, using mean MCP extension of non-dominant hand as the dependent variable. Because this was explorative and MRI parameters were not known previously, the sample size for MRI scanning was not hypothesis based. MRI quantitative descriptors are described as mean (standard deviation) for each group. Univariate and then multiple linear regression were used for structural determinants of hand stiffness (mean MCP angle of the imaged hand as the dependent variable, average skin thickness, average subcutaneous thickness, tenosynovial thickness, palmar fascia thickness, and median nerve area as predictor variables). We first standardized the independent and the dependent variables of the dataset into their corresponding Z-scores. All statistical analysis were performed using SPSS (IBM Corporation, Armonk, NY) and R (R Foundation, Vienna, Austria).

#### Ethics

This study received ethics permission from the KEM Hospital Research Centre Ethics Committee (KEMHRC/RVC/EC/1518), and patients signed separate informed consent forms for clinical examination and MRI scans. The study is registered with the Clinical Trials Registry of India (CTRI/2020/12/030057). Data sharing agreements were signed with Star Imaging and Research Centre and Indian Institute of Science Education and Research (IISER), Pune. The study received a waiver from the IISER Ethics Committee for Human Research (IEHCR/Admin/2021/007). All clinical and imaging data are stored at the Diabetes Unit, KEM Hospital Research Centre. Individual MRI results were made available to patients immediately; abnormalities found were offered treatment, such as perilesional steroids. Patient groups will be involved in disseminating the results of this study.

#### Role of funding

This study is funded through a DBT/Wellcome India Alliance Clinical and Public health fellowship (IA/CPHE/19/504607). The funding body had no role in study design or analysis.

## Results

### Cohort characteristics

We examined 237 adults with type 1 diabetes (90 male subjects, median age of 26.8 years). Fifteen (6%) patients were manual workers, and three patients were keyboard workers. The median duration of diabetes was 13.7 years; apart from insulin, 57 patients received metformin. One-fifth of the cohort (20 patients, 8.5%) had retinopathy, 28 (11.8%) patients had nephropathy, and 45 (19%) patients had neuropathy on clinical examination (**Table 1**).

**Table 1:** Patient characteristics at time of assessment.

Patient characteristics	n = 237
Male subjects (n, %)	90 (40.9%)
Age in years (median, range)	26.8 (20.4, 36.0)
Age at diagnosis, years (median, range)	12.3 (9.2, 16.4)
Duration of diabetes (median, range)	13.7 (8.1, 22.4)
Occupation: manual labor	15.0 (6.3%)
Occupation: keyboard >6 h	3.0 (1.3%)
Nicotine use, current or past	21.0 (8.9%)
Alcohol use, current or past	25.0 (10.5%)
Metformin use (n%)	57.0 (24.0%)
Retinopathy	20 (8.4%)
Proliferative	7
Non-proliferative	13
Nephropathy (n%)	28 (11.8%)
Neuropathy, clinical	45 (18.9%)
Exposure to additional fibrogenic disease or medication	34 (14.3%)
Weight (kg) (median, range)	56.8 (49.0, 66.1)
Height (cm, median, range)	160.0 (154.0, 169.0)
BMI (kg/cm^2^) (median, range)	22.0 (19.1, 24.9)
Lipohypertrophy	49 (20.7%)
Lipoatrophy	3 (1.3%)
Keloid scar	14 (5.9%)
AGE reading (median, IQR)	2.4 (1.9, 2.9)
Hand pain on the day of assessment	9 (3.8%)
Finger locking/triggering, current or past	26 (10.9%)
Paresthesia in hands, current or past	16 (6.8%)
Duruoz hand index (DHI), median	0
Duruoz hand index (DHI) >10 (n, %)	6 (2.5%)
Grip strength, dominant hand (mm Hg), median, IQR	41.7 (33.3, 57.5)
Grip strength, non-dominant hand (mm Hg), median, IQR	41.7 (33.3, 56.7)
Prayer sign (n%)	73.0 (30.8%)
Distance between fifth MCP, cm (mean, SD)	0.815 (3.01)
Distance between fifth PIP, cm (mean SD)	1.116 (3.10)
MCP extension angle, degrees - dominant hand (mean SD)	50.8 (16.7)
MCP extension angle, degrees - non-dominant hand (mean SD)	54.2 (16.9)
CTS (Tinel sign or Phalen sign or reduced/absent sensation)	53 (22.4%)
Physician diagnosis of diabetic cheiroarthropathy (n%)	79 (33.8%)
Dupuytren’s disease (n%)	3 (1.2%)
Flexor tenosynovitis (n%)	30.0 (12.6%)
Limited joint mobility (n%)	46.0 (19.4%)
CTS (n%)	24 (10.1%)
>1 condition	24 (10.1%)

BMI, body mass index; MCP, metacarpophalangeal; PIP, proximal interphalangeal; AGE, advanced glycation end-product; SD, standard deviation; IQR, interquartile range; CTS, carpal tunnel syndrome.

### Musculoskeletal history and hand examination

Only nine (3.8%) patients complained of hand pain; 26 patients had a history of trigger finger, and 16 (6.8%) patients had a history of paresthesia. Prayer sign was seen in 73 (30%) patients. Fifty-three patients had CTS on clinical examination. Seventy-nine (33.8%) patients had a cheiroarthropathy manifestation on physician’s assessment; 30, 46, 24, and 3 patients had FTS, LJM, CTS, and DC, respectively; 24 patients had more than one condition. Patients with a physician-diagnosed cheiroarthropathy had a significantly different average MCP extension angle (39° vs. 61°, p < 0.01) and a significantly higher DHI (1.67 vs. 0.21, p < 0.01).

### Patient characteristics as divided by MCP extension limitation

Mean extension at the MCP joint was 50.8° (SD) on the right hand and 54.2° on the left hand, with a range of 0° to 85° (**Table 2**). The largest group of patients (103, 43%) had mean extension in the range of 40° to 60°, whereas 64 (27%) patients had extension limited to less than 40°. The group with the most severe limitation (<20°) was predominantly male subjects (11/15, 73%), unlike all the other groups. Groups with limitation (0°–20° and 20°–40°) tended to be older and had diabetes for nearly a decade longer. One-fifth of the <20° group were manual workers; the group also had a much higher prevalence of smoking and/or alcohol use (nearly 30%) as compared with the other groups (approximately 10%). The group with the most severe restriction also had the highest prevalence of retinopathy (20%) and nephropathy (33%) but not neuropathy. Body composition did not vary across groups; subjects with MCP restriction had higher systolic blood pressure but not diastolic. They also had the highest prevalence of lipohypertrophy and keloid scar formation. Autofluorescence on AGE reader did not differ in the four groups.

**Table 2:** Clinical and MRI findings in patients grouped by degree of metacarpophalangeal joint mobility restriction.

Average MCP extension angle, nondominant hand categories (degrees)	0–20	20–40	40–60	>60	P for trend
Number	15	49	103	69	
Male subjects	11 (73.3%)	19.0 (38.8%)	45 (43.7%)	22 (31.9%)	0.02
Age, years (median, range)	33 (26, 44)	34 (23, 43.5)	25 (19, 33)	22 (19, 30)	0.00
Age at diagnosis, years (median, range)	13.5 (6.6, 14.4)	12 (8.8, 16.9)	12.4 (9.2, 15.9)	11.8 (9.3, 16)	0.51
Duration of diabetes, years (median, range)	23.9 (18.9, 33.6)	22.1 (13.2, 30.3)	11.4 (7.2, 18.7)	10.4 (7, 15.8)	0.00
Occupation: manual labor	3 (20.0%)	6 (12.2%)	4 (3.9%)	2 (2.9%)	
Occupation: keyboard workers	0	0	2 (1.9%)	1 (1.4%)	
Current or past smoking (n, %)	4 (26.7%)	4 (8.2%)	8 (7.8%)	5 (7.2%)	0.10
Current or past alcohol (n, %)	5 (33.3%)	3 (6.1%)	10 (9.7%)	7 (10.1%)	0.22
Metformin use (n, %)	5 (33.3%)	12 (24.5%)	20 (19.8%)	19 (27.5%)	0.50
Retinopathy (n, %)	3 (20.0%)	7 (4.3%)	7 (6.9%)	5.0 (7.4%)	0.06
Nephropathy (n, %)	5 (33.3%)	3.0 (6.1%)	14.0 (13.6%)	6.0 (8.7%)	0.11
Neuropathy, clinical (n, %)	2 (13.3%)	15.0 (30.6%)	19.0 (18.4%)	9.0 (13.0%)	0.11
Exposure to fibrogenic medication	5 (33.3%)	15 (30.6%)	10 (9.7%)	3 (4.3%)	0.00
Weight (kg) (median, range)	60.8 (44.6, 67.5)	58.2 (52, 69.2)	55.3 (49, 64.4)	56.6 (48, 65.4)	0.14
Height (cm) (median, range)	163 (150, 167)	159 (154.3, 168.8)	160.5 (153.4, 170)	160 (153.8, 169)	0.92
BMI (kg/cm^2^) (median, range)	21.9 (20.1, 24.7)	23.1 (20.5, 26.3)	21.5 (19, 24.8)	21.7 (18.7, 24.9)	0.05
Systolic BP (mm Hg) (median, range)	118 (110, 148)	122.5 (111, 139.8)	116 (109, 124.4)	114 (104.3, 120)	0.00
Diastolic BP (mm Hg) (median, range)	74.5 (67, 80)	76.5 (68.3, 84.5)	73.5 (67.3, 80)	72.5 (68, 79.9)	0.17
Lipohypertrophy (n%)	6 (40.0%)	11.0 (22.4%)	20.0 (19.4%)	12.0 (17.4%)	0.09
Lipoatrophy (n%)	0	0	2 (1.9%)	1 (1.5%)	0.53
Keloid scar (n%)	2 (13.3%)	2.0 (4.1%)	6 (5.8%)	4 (5.8%)	0.75
AGE reading (median, IQR)	2.4 (2.2, 2.7)	2.6 (2.2, 3.1)	2.2 (1.7, 2.8)	2.5 (2, 2.9)	0.17
Hand pain on day of exam (n, %)	1 (6.7%)	3 (6.1%)	4 (3.9%)	1 (1.5%)	0.17
Finger locking (n, %)	3 (20.%)	12 (24.5%)	7 (6.8%)	4 (5.8%)	0.00
Paresthesia (n, %)	2.0 (13.3%)	7.0 (14.3%)	5.0 (4.8%)	2.0 (3.0%)	0.01
Frozen shoulder (n, %)	3 (20.%)	12 (24.5%)	5 (4.8%)	0	0.00
Duruoz hand index (mean)	1.87	1.94	0.26	0.23	0.00
Duruoz hand index >10 (n%)	1.0 (6.7%)	3.0 (6.1%)	1.0 (0.97%)	1.0 (1.5%)	0.07
Grip strength, dominant hand (mm Hg), median, IQR	46.7 (36.7, 63.3)	43. 3 (32.5, 62.5)	43. 3 (33.3, 60.0)	40.0 (32.5, 48.3)	0.03
Grip strength, non-dominant hand (mm Hg), median, IQR	51.7 (40.0, 63.3)	41.7 (33.3, 63.3)	45.0 (33.3, 56.7)	40.0 (31.7, 46.7)	0.01
Prayer sign (n%)	12 (80.0%)	24 (48.9%)	10 (9.7%)	27 (39.1%)	0.00
Distance between both fifth MCP (cm) (mean, SD)	3.14 (4.54)	1.73 (5.77)	0.43 (0.93)	0.26 (0.66)	0.00
Distance between both fifth PIP (cm) (mean, SD)	5 (6.86)	2.15 (4.69)	0.64 (1.43)	0.29 (0.79)	0.00
MCP extension angle, degrees - dominant hand (mean, SD)	13 (7.1)	32.9 (5.2)	53.4 (5.8)	67.8 (5.5)	0.00
MCP extension angle, degrees - non-dominant hand (mean, SD)	16.2 (11.4)	36.6 (7.1)	57.4 (7)	70.1 (6.4)	0.00
Physician diagnosis of diabetic cheiroarthropathy (n%)	15 (100%)	35 (71.4%)	21.0 (20.4%)	7.0 (10.1%)	0.00
**Number of MRI scans**	**12**	**14**	**20**	**15**	**P for trend**
Mean tendon sheath thickness (mm)	0.58 (0.55, 0.61)	0.55 (0.46, 0.64)	0.5 (0.45, 0.55)	0.65 (0.52, 0.7)	0.821
Maximum tendon sheath thickness (mm)	0.7 (0.63, 0.88)	0.64 (0.55, 0.77)	0.56 (0.52, 0.63)	0.72 (0.56, 0.82)	0.1
Mean skin thickness (mm)	1.48 (1.1, 1.7)	1.4 (1.03, 1.6)	1.38 (1.09, 1.55)	0.95 (0.8, 1.25)	0.003
Mean subcutaneous thickness (mm)	2.58 (2.08, 3.48)	2.48 (2.25, 3.16)	2.15 (1.71, 2.74)	3.5 (2.5, 4.3)	0.211
Mean median nerve area (mm^2^)	8.95 (8.03, 9.9)	10 (8.88, 11.3)	8.6 (7.1, 9.1)	9 (6.88, 10.2)	0.076
Additive MRI fibrosis (mean skin thickness + mean tendon thickness + mean palmar facia thickness) (mean, SD)	2.40 (1.4)	2.02 (0.7)	1.87 (0.34)	1.64 (0.30)	0.011
Z-score addition score (mean, SD)	1.32 (3.37)	0.03 (1.65)	−0.48 (1.16)	−0.45 (1.1)	0.018

BMI, body mass index; MCP, metacarpophalangeal; PIP, proximal interphalangeal; AGE, advanced glycation end-product; SD, standard deviation; IQR, interquartile range.

Hand joint restriction was rarely painful: Only 7% complained of hand pain even in the most limited group. Similarly, although more often seen in the restricted groups, only a small fraction had symptoms (14%) of neuropathy. Both observations translated into a small but statistically significant higher DHI in the restricted groups (mean of 1.9 compared with that of 0.2). Only six percent in the <20° group had a DHI of more than 10, which we considered clinically relevant hand function restriction. Most patients (80%) were observed to have a prayer sign in the <20° group. All patients in the <20° group and three-fourths of the 20°–40° group had at least one described cheiroarthropathy phenotype on physician opinion. In univariate linear regression analyses adjusted for gender and duration of diabetes, height, systolic blood pressure, urine–albumin creatinine ratio/clinically determined nephropathy, and the presence of frozen shoulder were significantly associated with average non-dominant hand MCP extension angle (**Table 3**).

**Table 3:** Linear regression for factors contributing to metacarpophalangeal joint limitation.

	Model 1 (unadjusted)		Model 2 (adjusted for gender)		Model 3 (adjusted gender and duration of diabetes)	
	Std β	P-value	Std β	P-value	Std β	P-value
Weight	−0.074	0.257	−0.013	0.847	0.041	0.537
Height	0.021	0.753	0.262	0.003	0.212	0.01
Systolic BP	−0.305	0.000	−0.281	0.000	−0.203	0.002
Diastolic BP	−0.11	0.098	−0.128	0.051	−0.073	0.242
HbA1C	0.004	0.952	−0.005	0.939	−0.132	0.043
UACR	−0.164	0.024	−0.171	0.017	−0.204	0.002
Occupation (Manual Labor)	−0.071	0.28	−0.066	0.306	−0.003	0.967
Metformin	0.047	0.477	0.066	0.315	0.055	0.366
Retinopathy	−0.148	0.033	−0.144	0.036	−0.055	0.393
Nephropathy	−0.115	0.097	−0.099	0.15	−0.122	0.058
Neuropathy	−0.065	0.322	−0.088	0.179	−0.048	0.436
Fibrotic medication or disease	−0.242	0.000	−0.224	0.001	−0.205	0.001
Frozen shoulder	−0.258	0.000	−0.263	0.000	−0.182	0.004
Lipohypertrophy	−0.168	0.01	−0.173	0.007	−0.109	0.078
Lipoatrophy	0.045	0.494	0.031	0.631	0.025	0.681
Hypothyroidism	0.003	0.967	−0.033	0.616	0.026	0.683

BP, blood pressure; UACR, urine–albumin creatinine ratio.

### MRI findings

MRI scans of the hand were performed in 61 patients (12 in the <20° group, 14 in the 20°–40° group, 20 in the 40°–60° group, and 15 in the >60° group). The radiologist’s findings included flexor tendon thickening or edema in four, whereas one has tenosynovial edema in the abductor pollicis longus tendon (De-Quervain tenosynovitis). One patient had median nerve neuritis, and one had bifid median nerve. Eight patients had ganglion cysts, five of which in dorsal scapholunate ligament. One patient had early MCP degenerative changes; other MRI scans were reported as normal.

**Figure 2:**
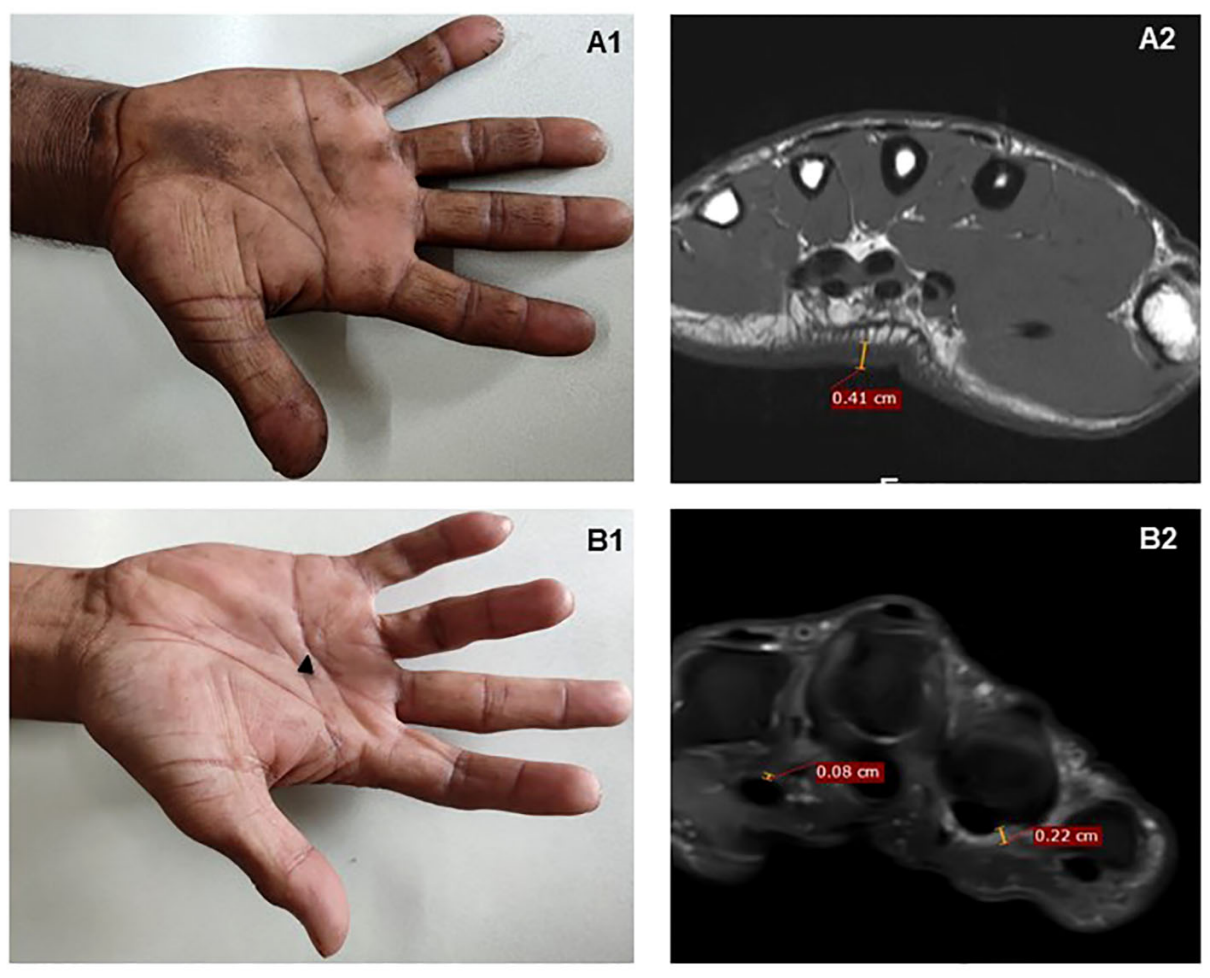
MRI findings in two patients with MCP extension restriction. **(A1)** A patient with severely restricted MCP extension – mean 10°. **(A2)** MRI demonstrates considerable palmar skin thickening alone on T1-weighted axial images, seen at mid-metacarpal level; there is no tenosynovial or palmar fascia thickening. **(B1)** A patient with bilateral Dupuytren’s contracture on the fourth finger (black arrow); **(B2)** PDFS scan shows ill-defined PDFS hyperintense soft tissue thickening in relation to flexor thickening also involving A1 pulley and the palmar fascia.

When analyzed by group, there was no significant difference within the groups in mean tendon sheath thickness, maximum tendon sheath thickness or median nerve area (**Table 2**). The group with the most stiffness had the highest skin thickness and reduced progressively; but subcutaneous thickness did not differ across groups (**Figure 2**). Both the total hand fibrosis score and the total hand fibrosis Z-score were able to differentiate between the four groups (**Table 2**).

Mean palmar skin thickness correlated significantly with palmar fascia thickness (correlation coefficient of 0.298, p = 0.02) but not with mean tenosynovial thickness (p = 0.25). Similarly, mean tenosynovial thickness also correlated with palmar fascia thickness (correlation coefficient of 0.28, p = 0.03). Univariate linear regression showed that only palmar skin thickness and palmar fascia thickness correlated with MCP angle restriction (**Table 4**). Only mean palmar skin thickness remained significantly associated with MCP angle in multiple linear regression (**Table 4**).

**Table 4:** Linear regression model for structural contributors to mean metacarpophalangeal extension on MRI.

	Model 1 (univariate)		Model 2 (multiple univariate)	
	Std β	P-value	Std β	P-value
Skin thickness	−0.376	0.003	−0.326	0.012
Mean subcutaneous thickness	0.182	0.161	–	
Palmar fascia thickness	−0.262	0.041	−0.165	0.193
Median nerve cross-sectional area	−0.243	0.064	–	
Tendon sheath thickness	−0.032	0.808	–	
R-Squared (R2)	–		13.7%	

## Discussion

One-third of an Indian cohort of adults with type 1 diabetes had diabetic cheiroarthropathy that was rarely symptomatic or functionally limiting but did substantially limit average MCP joint extension. The utility of the intuitively selected MCP extension limitation as a measure of hand stiffness is given plausibility by associations with structural correlates of fibrotic skin thickening on MRI. Expected features such as tenosynovial inflammatory edema or median nerve enlargement were rare. An additive score of tissue thickening was able to differentiate between levels of joint stiffness. Apart from novel insights into the patho-anatomy of hand stiffness, our data suggest a framework for quantification of hand fibrosis regardless of individual cheiroarthropathy manifestation, both on clinical examination and imaging. After further validation, they could be used as metrics for associative studies with other profibrotic manifestations and interventions.

Although cheiroarthropathy is often reported in both type 1 and type 2 diabetes, its implications in clinical practice are yet inconclusive. Age and duration of diabetes are pervasively associated, regardless of definitions and population (2, 7, 9). An older age and longer duration of the cohort explains the near double prevalence found in the Epidemiology of Diabetes Interventions and Complications/Diabetes Control and Complications Trial cohort as well as a Danish patient registry (2, 21) LJM prevalence reduced from 43% to 23% in adult patients with type 1 diabetes over two decades (7). Reduced prevalence has been attributed to better glycemic care; however, the association of hand manifestations with hemoglobin A1C is inconsistent (22). The pathobiology in the ones who do have hand manifestations is intriguing. Associations with microvascular disease including retinopathy are reported (2, 23) but not always replicated (9). Could hand stiffness demonstrate a profibrotic tendency not entirely explained by vascular disease? (14) We found some associative signals with systolic blood pressure, proteinuria, and adhesive capsulitis; each a proxy for arterial, renal, and musculoskeletal fibrosis, respectively, but not with other microvascular disease. Similar associations with nephropathy have been found with MCP and wrist flexion angles (18). Future studies of associations with markers of fibrotic liver and cardiac disease would be informative if the hand could be used as a clinical biomarker of internal fibrosis.

Although hand stiffness was common, it was rarely symptomatic or functionally limiting, thus making the case for active screening by a physician. Painful hand conditions are often encapsulated by functional indices such as the Health Assessment Questionnaire (HAQ); Disabilities of the Arm, Shoulder, and Hand (DASH); and the DHI (24–26). The higher DHI seen in our study in the stiffer group would not be considered clinically significant (20). Other studies echo the insubstantial functional impact of these conditions using different scores such as HAQ and the DASH score (2, 27). Only 10% of patients with diabetes volunteered symptoms in the hand, showing wide discrepancy in patient-volunteered symptoms and physician-examined manifestations (8). Symptomatic patients likely represent a subset with advanced stiffness; a quantitative metric would be useful if it can detect early manifestations, especially in irreversible but potentially preventable process like fibrosis. Most descriptions of joint stiffness use the table top test or prayer sign (28). Goniometry offers a more reproducible, quantitative metric. Using goniometry, authors showed that, although most joints assessed were less flexible in diabetics, the MCP and distal IP joints were most severely affected (29). We selected average resistance to MCP stretch as a metric that would intuitively encapsulate stiffness regardless of specific manifestation: Whereas LJM affects all fingers, DC and FTS may affect one or multiple digits. All of these would reduce mean MCP extension, but DC and FTS are not expected to reduce wrist extension. Although we did not check for MCP flexion, it is likely to be far less affected as attested by normal grip strengths; average MCP flexion was well preserved in a type 1 longitudinal cohort, with only a 10° loss over 15 years (18). Although ours is not a longitudinal study, the range of LJM extension was wide.

Previous MRI descriptions of diabetic cheiroarthropathy are limited to a single case report that showed flexor tenosynovial sheath thickening on T2-weighted images and tenosynovial proliferation on axial T1 fat saturation gadolinium enhancement (30). Similarly, flexor tendon sheath and subcutaneous tissue thickening were found on ultrasound in diabetic cheiroarthropathy (31). We found that skin thickening contributed most to LJM; these may be explained by differences in patient selection, population differences, and diagnostic modality. A notable feature in our cohort was the relatively normal size of the median nerve, suggesting that patients with type 1 diabetes may not have the expected increase in median nerve cross-sectional area routinely used to diagnose CTS (32). Median nerve cross-sectional area was smaller in diabetics with CTS (mean of 8.8 mm^2^) than with patients with CTS without diabetes (mean of 10.4 mm^2^) (33). Future work in conjunction with nerve conduction velocities is useful in determining whether patients with diabetes need separate cutoff values for MRI diagnosis of CTS.

Regardless of the degree of hand stiffness, inflammation on MRI was conspicuous by its absence, contrasting with MRI findings in FTS in rheumatoid arthritis (34) and systemic sclerosis (35). A small number of our patients, like the one in the previous case report, had tenosynovial edema (30). It is possible that the time of assessment along the temporal evolution of the manifestation makes a difference, with an inflammatory initiation continuing to fibrosis. Most inflammatory tenosynovitis cases are painful, although pain was rare in our group (34). It could be postulated that non-inflammatory profibrotic pathways such as AGE deposition and hypoxia might be more important disease mechanisms.

Our study has many strengths: To our knowledge, it is the first systematic evaluation of joint stiffness in type 1 diabetes, and the structural differences on MRI provide credence to MCP extension angle as a quantifiable clinical measure of hand fibrosis. Both clinical measurements and MRI measurements were performed by more than one assessor, suggesting a reproducibility of methods.

This study has limitations: Even the “controls” with no hand stiffness were diabetic, and future work would include age-matched non-diabetic controls. We restricted this study to type 1 diabetes, despite the hand conditions being seen in both types. We reckoned that patients with type 1 diabetes would have fewer clinical confounders, including age, medications with antifibrotic properties (such as metformin and pioglitazone), and accrual of damage with manual work. Future work would encompass both types of diabetes as well as prediabetes. We did not compare the performance of MCP extension to other joints. The groupings of 20° were arbitrarily decided on the basis of perceived convenience for a clinician, and each group had unequal numbers on MRI. The sample for imaging was not calculated statistically, because a goniometer was not used to measure MCP extension angles; however, the lack of specialized equipment would, in our opinion, make the screening measurement easier to perform in the community.

In conclusion, we found a substantial subset of patients with adult type 1 diabetes had diabetic cheiroarthropathy and resulting limitations in MCP extension. Joint mobility limitation was rarely symptomatic or functionally limiting, warranting an active search in the clinic. Joint stiffness was driven by skin thickening, and inflammatory tenosynovial involvement was rare on MRI. Our data provide a framework for a quantitative assessment of hand fibrosis even in subclinical disease, using mean MCP extension in the clinic, and using additive tissue thickness scores on MRI. Pending more extensive validation, these measurements could potentially be used to elucidate associations with internal organ fibrosis, as well as for sample size calculations and outcome measures for trials for musculoskeletal fibrosis.

## Preprint

A version of this article has been posted on the medRxiv preprint server.

## Guarantor statement

SP is the guarantor of this work and, as such, had full access to all the data in the study and takes responsibility for the integrity of the data and the accuracy of the data analysis.

## Data availability statement

The raw data supporting the conclusions of this article will be made available by the authors, without undue reservation.

## Ethics statement

The studies involving humans were approved by KEM Hospital Research Centre Ethics Committee. The studies were conducted in accordance with the local legislation and institutional requirements. The participants provided their written informed consent to participate in this study. Written informed consent was obtained from the individual(s) for the publication of any potentially identifiable images or data included in this article.

## Author contributions

SP, JI, PG, and CS were involved in the conception and design of the study. SC, SaJ, RS, and SP were involved in the conduct of the study. SP, KC, SC, SwJ, and AB were involved in analysis and interpretation of the results. SP and KC wrote the first draft of the manuscript. SP is the guarantor of this work and, as such, had full access to all the data in the study and takes responsibility for the integrity of the data and the accuracy of the data analysis. All authors contributed to the article and approved the submitted version.
