# Corrigendum: Quantification of joint mobility limitation in adult type 1 diabetes

**DOI:** 10.3389/fendo.2023.1339588

**Published:** 2023-12-14

**Authors:** Sanat Phatak, Pranav Mahadevkar, Kaustubh Suresh Chaudhari, Shreya Chakladar, Swasti Jain, Smita Dhadge, Sarita Jadhav, Rohan Shah, Aboli Bhalerao, Anupama Patil, Jennifer L. Ingram, Pranay Goel, Chittaranjan S. Yajnik

**Affiliations:** ^1^ Diabetes Unit, King Edward Memorial (KEM) Hospital Research Centre, Pune, India; ^2^ Department of Musculoskeletal Radiology, Star Imaging and Research Centre, Pune, India; ^3^ Department of Biology, Indian Institute of Science Education and Research, Pune, India; ^4^ Division of Pulmonary, Allergy and Critical Care, Department of Medicine, Duke University Medical Center, Durham, NC, United States

**Keywords:** magnetic resonance imaging (MRI), outcome measure (healthcare), tenosynovitis, metacarpophalangeal (MCP) joint, limited joint mobility (LJM), stiffness

In the published article, there was an error in the Funding statement. The Funding statement acknowledged the funders of this work, however other funders have been supporting patient care. Thus, three philanthropic donors have been added to the statement: Hinduja Foundation, Mukul Madhav Foundation, and Nityasha. The correct Funding statement appears below.

This study is funded through a DBT/Wellcome India Alliance Clinical and Public health fellowship (IA/CPHE/19/504607) awarded to SP. The funding body had no role in study design or analysis. Many participants with type 1 diabetes in this study are beneficiaries of financial and social support from a ‘type 1 diabetes initiative’ of the following philanthropic donors: 1) Hinduja Foundation, 2) Mukul Madhav Foundation, 3) Nityasha. The authors are grateful to these organisations for their continued support in the management of people with type 1 diabetes.

The authors apologize for this error and state that this does not change the scientific conclusions of the article in any way. The original article has been updated.

